# Structure of Ldt_Mt2_, an l,d-transpeptidase from *Mycobacterium tuberculosis*

**DOI:** 10.1107/S0907444912049268

**Published:** 2013-02-16

**Authors:** Dominic Böth, Eva Maria Steiner, Daniela Stadler, Ylva Lindqvist, Robert Schnell, Gunter Schneider

**Affiliations:** a Department of Medical Biochemistry and Biophysics, Karolinska Institutet, S-17 177 Stockholm, Sweden

**Keywords:** *Mycobacterium tuberculosis*, cell wall, peptidoglycans, transpeptidases, antibiotics

## Abstract

The transpeptidase Ltd_Mt2_ catalyzes the formation of the (3–3) cross-links characteristic of the peptidoglycan layer in the *Mycobacterium tuberculosis* cell wall. Bioinformatics analysis suggests that the extramembrane part of the enzyme consists of three domains: two smaller domains (denoted as A and B domains) and a transpeptidase domain (the C domain) at the C-­terminus. The crystal structures of two fragments comprising the AB domains and the BC domains have been determined. The structure of the BC module, which was determined to 1.86 Å resolution using Se-SAD phasing, consists of the B domain with an immunoglobulin-related fold and the catalytic domain belonging to the ErfK/YbiS/YbnG fold family. The structure of the AB-domain fragment, which was solved by molecular replacement to 1.45 Å resolution, reveals that despite a lack of overall sequence identity the A domain is structurally very similar to the B domain. Combining the structures of the two fragments provides a view of the complete three-domain extramembrane part of Ldt_Mt2_ and shows that the protein extends at least 80–100 Å from the plasma membrane into the peptidoglycan layer and thus defines the maximal distance at which cross-links are formed by this enzyme. The Ldt_Mt_-related transpeptidases contain one or two immunoglobulin domains, which suggests that these might serve as extender units to position the catalytic domain at an appropriate distance from the membrane in the peptidoglycan layer.

## Abstract 2

The crystal structures of two fragments of the l,d-transpeptidase from *M. tuberculosis* have been determined at 1.45 and 1.86 Å resolution. The extramembrane part of this enzyme consists of three domains: two domains related to the immunoglobulin fold and a catalytic domain belonging to the ErfK/YbiS/YhnG family at the C-terminus.

## 1. Introduction   

The cell wall of *Mycobacterium tuberculosis* is a complex multilayered structure with a unique architecture that protects the organism against both physical and chemical stress (Hett & Rubin, 2008 ▶). This barrier supports the survival of this pathogen in host macrophages and confers resistance to many antibiotics (Hett & Rubin, 2008 ▶; Gengenbacher & Kaufmann, 2012 ▶; Russell, 2011 ▶). The increasing number of multidrug-resistant (MDR-TB) and extensively drug-resistant (XDR-TB) strains of *M. tuberculosis* makes treatment using current strategies increasingly difficult (Cole & Riccardi, 2011 ▶). Since *M. tuberculosis* expresses a significant number of class A β-­lactamases (Flores *et al.*, 2005 ▶; Hugonnet & Blanchard, 2007 ▶), β-lactams are not considered to be a promising front-line medication against tuberculosis. However, interest in the treatment of tuberculosis by targeting the mycobacterial cell wall using a combination therapy of potent lactamase inhibitors of the clavulanate family and carbapenem-type β-­lactams has been revived. For instance, the clavulanate–carbapenem combination has shown a bactericidal effect against dormant mycobacteria and drug-resistant strains and in a mouse tuberculosis model (Hugonnet *et al.*, 2009 ▶; England *et al.*, 2012 ▶).

In the complex mycobacterial cell wall, the peptidoglycan (PG) layer is responsible for rigidity and osmotic stability and provides the foundation of the cell envelope (Hett & Rubin, 2008 ▶). The mycolic acids and glycolipids that form a wax-like coat in the outer layer are covalently linked to arabino­galactan, which in turn is coupled to *N*-acetylmuramic acid units in the PG (Brennan & Crick, 2007 ▶). PG is a complex polymer built up of a glycan chain of alternating units of *N*-­acetylglucosamine and *N*-acetylmuramic acid and short oligopeptide stems coupled to the *N*-acetylmuramic acid units *via* a lactate moiety. The peptide stems are cross-linked, connecting different glycan strands into a meshwork that builds up the PG layer surrounding the cell membrane (Vollmer *et al.*, 2008 ▶; Meroueh *et al.*, 2006 ▶). The integrity of the peptide cross-links is vital for bacteria and the enzymes involved in the formation of these linkages are targeted by β-­lactam antibiotics. The amino-acid composition and cross-link architecture show large variations when comparing different bacterial species. *M. tuberculosis* displays a common peptide-stem sequence (l-Ala-d-γGlu-Dap-d-Ala) which is also found in *Escherichia coli* and *Bacillus subtilis*, but with characteristic modifications. These are the high level of amidation on the d-glutamate (d-Glu) and diaminopimelate (Dap) moieties and a high proportion (80%) of Dap–Dap (3–3) cross-links (Kumar *et al.*, 2012 ▶; Lavollay *et al.*, 2008 ▶). The peptide linkages are produced by periplasmic transpeptidases that are active in PG polymerization (Lovering *et al.*, 2012 ▶). The common Dap-d-Ala (3–4) cross-links are formed by d,d-­transpeptidases, which are also called penicillin-binding proteins (PBPs). In contrast, the Dap–Dap (3–3) cross-links are created by l,d-transpeptidases (Mainardi *et al.*, 2005 ▶), which represent a different class of enzymes that are not related to the d,d-transpeptidases (Biarotte-Sorin *et al.*, 2006 ▶; Bielnicki *et al.*, 2006 ▶). l,d-Transpeptidases are also responsible for the coupling of nonconventional d-amino acids to PG peptide stems, which has been suggested to be a stress-induced cell-wall adaptation in *Vibrio cholerae* (Cava *et al.*, 2011 ▶).

The *M. tuberculosis* H37Rv genome encodes five proteins that all contain a characteristic l,d-transpeptidase domain (Rv0116c, Rv0192, Rv0483, Rv1433 and Rv2518c). Ldt_Mt2_ (Rv2518c) has been reported to be expressed at the highest level and has been found to be essential for virulence. In­activation of this gene led to an attenuated phenotype and to increased susceptibility to clavulanate/amoxicillin treatment *in vitro* and in a mouse tuberculosis model (Gupta *et al.*, 2010 ▶). The importance of Ldt_Mt2_ in virulence makes it an attractive target for the development of inhibitors and novel antibiotics. Structurally characterized l,d-transpeptidases from *B. subtilis* (Bielnicki *et al.*, 2006 ▶) and from *Enterococcus faecium* (Biarrotte-Sorin *et al.*, 2006 ▶) define the architecture of the catalytic domain. However, both display an additional domain unrelated to the approximately 200-amino-acid long segment preceding the catalytic domain in Ldt_Mt2_ that may govern the positioning of the catalytic module in the PG layer of the cell wall.

Here, we report the three-dimensional structure of Ldt_Mt2_ (l,d-transpeptidase 2 from *M. tuberculosis*) based on the X-­ray crystallographic structures of two fragments of Ldt_Mt2_ representing the extramembrane part of the protein (residue range 55–408). The structure analysis reveals that Ldt_Mt2_ folds into three domains: two domains in the N-terminal part, both of which show an immunoglobulin-related fold, and a C-­terminal transpeptidase domain. This domain composition is different from the two-domain structure of the extramembrane part of Ldt_Mt2_ proposed recently (Erdemli *et al.*, 2012 ▶). The crystal structures of the Ldt_Mt2_ constructs allow modelling of the full-length extramembrane part of the enzyme (residues 55–408), providing an estimate of the maximal distance of the catalytic site from the membrane and thereby the approximate distance at which 3–3 cross-links are formed in the PG layer. Mass-spectrometric analysis provides further evidence that Ldt_Mt2_ forms covalent adducts with the β-lactam antibiotics imipenem and ampicillin.

## 2. Materials and methods   

### 2.1. Gene expression and protein purification   

**Figure 1:**
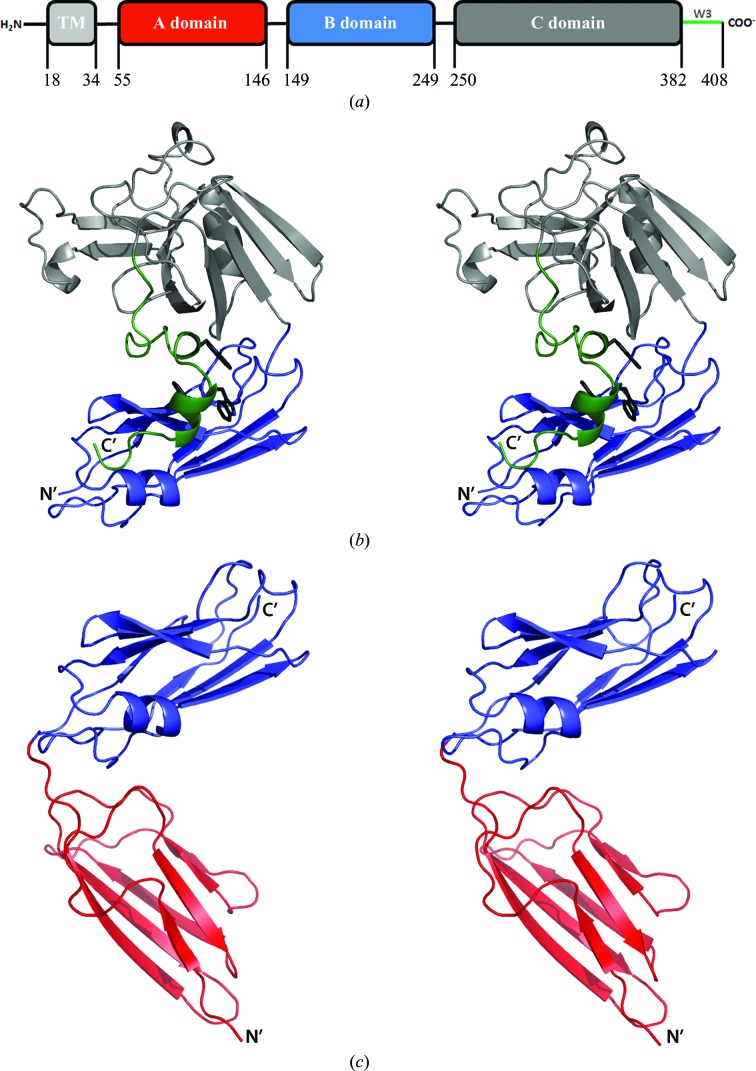
(*a*) Domain arrangement in the extramembrane segment of the l,d-­transpeptidase Ldt_Mt2_ from *M. tuberculosis*. TM, transmembrane helix. (*b*) Stereo cartoon of the structure of the BC module of Ldt_Mt2_. The catalytic domain is shown in grey and the B-domain in blue. The C-­terminal extension that folds over the B domain is shown in green. The three tryptophan residues within this segment are shown as black sticks. (*c*) Stereo cartoon of the structure of the AB module of Ldt_Mt2_. The two immunoglobulin-like domains are shown in blue (B domain) and red (A domain).

The expression construct for the complete extramembrane segment of Ldt_Mt2_ (UniProt code O53223; Rv2518c) included amino acids 34–408, lacking only the predicted transmembrane domain (residues 18–34) and the intracellular part of the polypeptide chain (residues 1–17). Additional constructs were based on the domain arrangement obtained from a bioinformatics analysis of the protein sequence (see §[Sec sec3]3). The construct used in structure determination comprising the two N-terminal domains A and B (the AB module) included residues 55–250, while the construct comprising the B and C domains (the BC module) encoded residues 149–408 (Fig. 1 ▶ *a*).

The coding sequences for these constructs were amplified by PCR using *Pfu* Turbo polymerase (Stratagene) with *M. tuberculosis* H37Rv genomic DNA as the template. Each construct was expressed in the pNIC28Bsa4 vector (GenBank accession No. EF198106) with a cleavable N-terminal His_6_ tag (H_2_N-MHHHHHHSSGVDLGTENLYFQ*SM). Proteolytic tag removal by TEV protease (the cleavage position is indicated by an asterisk) resulted in recombinant proteins with two additional amino acids (Ser-Met) at the N-terminus. All proteins were expressed in *E. coli* BL21 (DE3) cells at 294 K in LB medium supplemented with 30 µg ml^−1^ kanamycin. Gene expression was induced by the addition of 0.1 m*M* IPTG at an OD_600_ of 0.5–0.6 and the cells were harvested 20 h post-induction. The pellets were washed in 0.3 *M* NaCl, 10 m*M* Tris–HCl pH 8.0 and the cell walls were disrupted by flash-freezing and treatment with lysozyme (40 µg ml^−1^) and DNase I (2 µg ml^−1^) followed by sonication. The recombinant proteins were purified by affinity chromatography using His-Pur Ni–NTA resin (Thermo Scientific) and were eluted with an increasing imidazole gradient. Subsequently, the buffer was exchanged to 0.15 *M* NaCl, 25 m*M* Tris–HCl pH 8.0 using a desalting column (HiPrep 26/10; Pharmacia Biotech). The His_6_ tag was removed by TEV protease cleavage with 5 µg TEV per milligram of recombinant protein at 293 K in the presence of 2 m*M* DTT. Cleaved proteins were separated from residual uncleaved proteins and TEV protease by a second Ni–NTA affinity step. Finally, a size-exclusion chromatography step was performed using a Superdex 200 column (Pharmacia Biotech) equilibrated with a buffer consisting of 0.15 *M* NaCl, 25 m*M* Tris–HCl pH 8.0. Pure protein samples were concentrated using a Vivaspin 20 centrifugal device (Sartorius, Göttingen, Germany) and the protein concentration was determined according to the Bradford assay using reagents from Thermo Scientific (Rockford, Illinois, USA) and a BSA standard. The samples were analyzed by SDS–PAGE and native PAGE (PHAST system, Pharmacia Biotech), aliquoted in the final buffer (0.15 M NaCl, 25 m*M* Tris–HCl pH 8.0), flash-frozen in liquid nitrogen and stored at 193 K. The selenomethionine-substituted BC-module construct was produced *via* the metabolic inhibition method (Doublié, 1997 ▶) and purification was carried out using the same steps as for the native protein.

### 2.2. Protein crystallization   

Protein crystallization screening was carried out at protein concentrations of 17.5 mg ml^−1^ for the construct comprising the B and C domains and 23.2 mg ml^−1^ for the construct comprising the A and B domains by the vapour-diffusion method using a Mosquito Crystallization Robot (TTP LabTech Ltd, Melbourn, England) and the commercially available crystallization screens PACT, JCSG+, NeXtal Classics II Suite (Qiagen) and SaltRX (Hampton Research). Crystals of the AB module were obtained in sitting drops at 293 K by mixing 0.15 µl protein solution and 0.15 µl well solution [0.2 *M* ammonium sulfate, 0.1 *M* bis-tris–HCl pH 6.5, 25%(*w*/*v*) PEG 3350] and equilibrating against 50 µl well solution. The crystal used for data collection was flash-cooled in liquid nitrogen without using a cryoprotectant. The crystals of the BC module were grown in a hanging-drop format at 293 K by mixing 1 µl protein solution and 2 µl well solution (3 *M* sodium acetate pH 6.0, 0.1 *M* bis-tris propane–HCl pH 7.0) and equilibrating against 1.0 ml well solution. Crystals of the selenomethionine-substituted BC module were obtained in conditions identical to those for the native protein. The crystals of the BC module (native and selenomethionine-substituted) were cryoprotected by a short soak (20 s) in mother liquor supplemented with 30%(*v*/*v*) ethylene glycol prior to freezing. The crystals were frozen in nylon loops and stored in liquid nitrogen until data collection.

### 2.3. Data collection   

X-ray data sets were collected to 1.45 Å resolution for the AB module and to 1.86 Å resolution for the BC module on beamline ID14-1 of the European Synchrotron Radiation Facility (ESRF), Grenoble, France. In addition, a data set was collected to 2.7 Å resolution from a crystal of the SeMet-substituted BC module on beamline ID29 at the ESRF at the Se edge at a wavelength of 0.97677 Å. All data sets were collected at 100 K. The X-ray data were processed and scaled using *XDS* (Kabsch, 2010 ▶; AB module) and *MOSFLM* (Leslie, 2006 ▶; BC module) and scaled with *SCALA* from the *CCP*4 suite (Winn *et al.*, 2011 ▶).

The crystals of the AB module belonged to space group *C*2, with unit-cell parameters *a* = 114.4, *b* = 27.6, *c* = 68.0 Å, β =114.1°, and contained one molecule in the asymmetric unit with a solvent content of 47.8%. Crystals of the BC module (native and selenomethionine-substituted) belonged to space group *I*2_1_2_1_2_1_, with unit-cell parameters *a* = 116.0, *b* = 121.4, *c* = 123.3 Å, and contained two molecules in the asymmetric unit with a solvent content of 68%. The statistics for all data sets are summarized in Table 1 ▶.

### 2.4. Structure determination and refinement   

The structure of the BC module was solved by Se-SAD phasing using the data sets obtained from crystals of the SeMet-substituted protein. The Se sites were identified and refined from the peak data set using *SHELX* (Sheldrick, 2008 ▶). Of the 12 selenium sites in the asymmetric unit, 11 were found by *SHELX*, with CC values of 47.2% (CC_all_) and 30.1% (CC_weak_). An almost complete model was built by *ARP*/*wARP* (Mooij *et al.*, 2009 ▶) using the SeMet peak data set. From this point, the 1.86 Å resolution native data set was used to complete the model by iterative rounds of manual model building using *Coot* (Emsley *et al.*, 2010 ▶) and crystallographic refinement with *REFMAC*5 (Murshudov *et al.*, 2011 ▶) applying local NCS symmetry and TLS. The final model contained two chains of the BC module comprising protein residues 149–408, 12 acetate ions, two Na^+^ ions and 480 water molecules. The final crystallographic *R* and *R*_free_ values were 16.5% and 18.7%, respectively (Table 1 ▶).

The structure of the AB module was solved by molecular replacement using *Phaser* (McCoy *et al.*, 2007 ▶) with the B domain (149–250) derived from the BC module as the search model. Density modification using *Parrot* (Cowtan, 2010 ▶) was employed to improve the electron-density map, in particular for the unknown domain A at the N-­terminus. The A domain was built manually using *Coot* (Emsley *et al.*, 2010 ▶) and refined to 1.45 Å resolution by cycles of restrained refinement using *REFMAC*5 (Murshudov *et al.*, 2011 ▶). The final structure comprised amino-acid residues 57–250, two sulfate ions and 309 water molecules; the crystallographic *R* and *R*_free_ values were 16.2% and 21.8%, respectively (Table 1 ▶).

Both protein models were validated using *Coot* (Emsley *et al.*, 2010 ▶) and *MolProbity* (Chen *et al.*, 2010 ▶) to monitor the stereochemistry and model quality. A summary of the refinement statistics and model parameters is given in Table 1 ▶. Structural comparisons were carried out using the *DALI* algorithm (Holm & Rosenström, 2010 ▶; Holm & Park, 2000 ▶). Molecular contacts and interacting surfaces were analyzed using the *PISA* server (Krissinel & Henrick, 2007 ▶). Figures were produced using the program *PyMOL* (http://www.pymol.org). The coordinates of the AB module and BC module have been deposited in the Protein Data Bank under accession codes 4hu2 and 4huc, respectively.

### 2.5. Mass-spectrometric analysis of antibiotic binding   

Samples of Ldt_Mt2_ (5 µg) were dispensed in water without antibiotics and with 2 m*M* imipenem (Sigma) or ampicillin (Duchefa Biochemie, Haarlem, The Netherlands) and incubated for 45 min at 295 K. Subsequently, the samples were diluted in 0.5 ml denaturing buffer [5%(*v*/*v*) acetonitrile, 0.1%(*v*/*v*) formic acid, 0.5 m*M* TCEP] and loaded onto a CapLC system and an ESI-Q-TOF quadrupole/orthogonal acceleration time-of-flight mass spectrometer (Waters Corp.) using the method described by Sundqvist *et al.* (2007 ▶). The spectra were combined and deconvoluted with the *Maximum Entropy* 1 algorithm from the *MassLynx* software suite (Waters Corp.) to obtain the molecular weights of the protein molecules and the protein–antibiotic adducts.

## 3. Results and discussion   

### 3.1. Domain organization of Ldt_Mt2_, construct design and protein production   

The *lppS* gene (Rv2518c) encodes an enzyme consisting of 408 amino acids denoted Ldt_Mt2_. The polypeptide chain contains a short intracellular segment (residues 1–17), a predicted transmembrane domain (residues 18–34) and a larger extramembrane part (residues 35–408). The amino-acid sequence of Ldt_Mt2_ was analyzed with respect to potential domain organization using transmembrane-domain prediction (Cserzö *et al.*, 1997 ▶) and sequence comparisons by *BLAST* (Altschul *et al.*, 1990 ▶) and *ClustalW* (Thompson *et al.*, 1994 ▶). This resulted in a well defined prediction of the catalytic domain (residues 250–408). The sequence similarity of residues 150–250 (30–40% identity) to three other *M. tuberculosis* proteins (Rv0116c, Rv0483 and Rv1433) and several proteins derived from different corynebacteria genomes that also contain a predicted l,d-transpeptidase domain suggested that this fragment may be a separate domain. Furthermore, the sequence comprising residues 34–250 was subjected to domain-border analysis/prediction (http://www.tuat.ac.jp/~domserv/cgi-bin/DLP-SVM.cgi#Wahtis; Ebina *et al.*, 2009 ▶) as well as to secondary-structure prediction by *JPred* (Cole *et al.*, 2008 ▶) to define the domain borders. Finally, two likely starting points at the N-terminus were the end of the predicted transmembrane helix coinciding with a predicted lipoprotein-attachment site (Rahman *et al.*, 2008 ▶) at position 34/35 and the beginning of the first predicted secondary-structure element at position 55. Taken together, these analyses suggested that the extramembrane part of the enzyme consists of three domains: two smaller domains (domain A, residues 34/55–146; domain B, residues 149–250) and a catalytic domain (domain C, residues 250–408) belonging to the ErfK/YbiS/YhnG family (Bielnicki *et al.*, 2006 ▶; Fig. 1 ▶ *a*). Based on this analysis, three constructs were designed: a full-length construct (residues 34–408), the AB module (residue 55–146) and the BC module (residues 149–408).

The full-length extramembrane fragment was expressed in *E. coli* and purified to homogeneity. Based on size-exclusion chromatography, the protein is a monomer in solution. However, extensive crystallization screens did not result in crystal formation. The AB- and BC-module constructs also resulted in soluble protein and each construct behaved as a monomer in solution.

### 3.2. The structure of the BC module   

**Figure 2:**
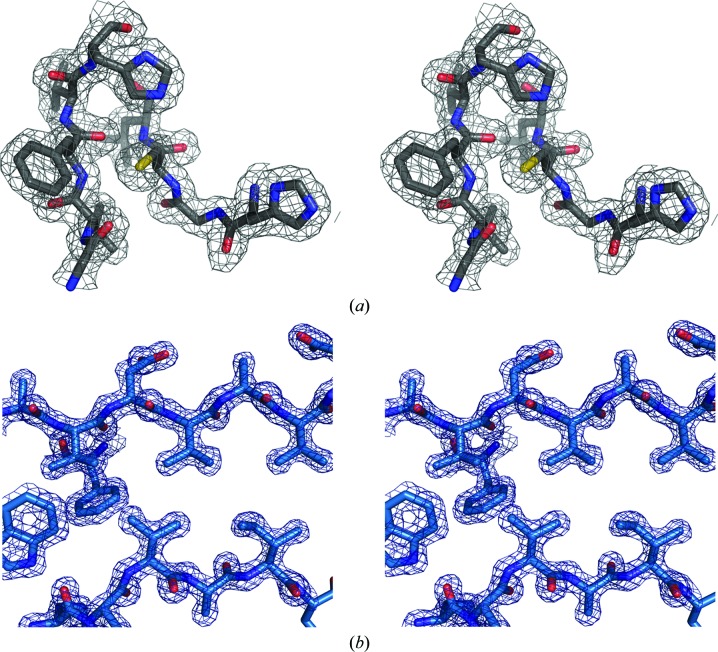
Parts of the refined 2*F*_o_ − *F*_c_ electron-density maps of Ldt_Mt2_ contoured at 1.5σ. (*a*) Stereoview of the electron-density map of the BC module in the vicinity of the active-site residue Cys354. (*b*) Stereoview of the electron-density map of the AB module in the hydrophobic core of the B domain.

The crystal structure of the BC module was solved to 1.86 Å resolution by Se-SAD phasing from crystals of selenomethionine-substituted protein. The electron density for the two polypeptide chains (residues 149–408) in the asymmetric unit is well defined (Fig. 2 ▶ *a*). Six surface residues were modelled in double conformations (Arg297 and Ile301 in chain *A* and Met237, His246, Arg297 and Lys370 in chain *B*). The model also contains 480 water molecules and 12 acetate ions, which mostly interact with surface residues. Two of the acetate ions are located in the active-site cleft of domain C. Finally, each polypeptide chain contains a bound metal ion. We modelled the bound metal as an Na^+^ ion based on the electron density (*i.e.* no remaining difference density after refinement), typical metal–ligand distances in the range 2.2–2.5 Å and the composition of the ligand sphere. The Na^+^ ion is located at the C-­terminus of a surface helix and is coordinated by the carbonyl O atoms of residues 342 and 345 and by three water molecules.

The BC module consists of the catalytic domain of the typical ErfK/YbiS/YhnG fold and a smaller domain with an immuno­globulin-related fold (Fig. 1 ▶ *b*). The crystal asymmetric unit contains two molecules with a monomer–monomer interface of approximately 800 Å^2^, suggesting crystal-packing contacts rather than dimer formation. The structures of the two molecules in the asymmetric unit in the crystals of the BC module are essentially identical, displaying a C^α^ r.m.s.d. of 0.3 Å for 259 aligned residues. The overall structure of this module and the interactions of the two polypeptide chains in the asymmetric unit are very similar to a recently determined structure of a corresponding module of Ldt_Mt2_, albeit obtained from a different expression construct (residues 120–408 as judged from Fig. 1*b* in Erdemli *et al.*, 2012 ▶; the atomic coordinates were not available in the PDB).

**Figure 3:**
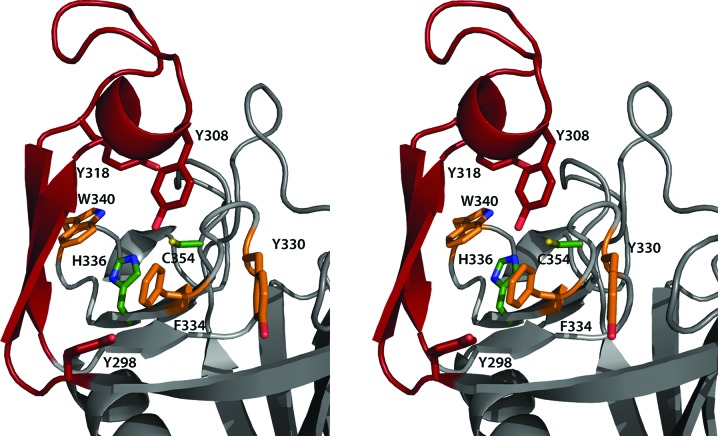
The active site of Ldt_Mt2_. The lid covering the active site formed by a pair of antiparallel β-strands and their connecting loop (residues 298–324) is shown in red. The active-site residues Cys354 and His336 are depicted as green sticks. The bulky hydrophobic residues of the lid (Tyr298, Tyr308 and Tyr318) and its surroundings (Tyr330, Phe334 and Trp340), shown as red and orange sticks, respectively, limit access to and contribute to the closure of the active site.

The core of the transpeptidase domain consists of a β-­sandwich formed by two β-sheets packed together. Comparison of this domain with a related protein from *E. faecium* (PDB entry 1zat; Biarrotte-Sorin *et al.*, 2006 ▶) using *DALI* results in a *Z*-score of 19.3 and a C^α^ r.m.s.d. of 1.9 Å for 120 aligned residues. A similar comparison with the *B. subtilis* homologue (PDB entry 1y7m; Bielnicki *et al.*, 2006 ▶) gives a *Z*-­score of 17.7 and a C^α^ r.m.s.d. of 1.6 Å for 109 aligned residues. The key residues of the catalytic site, Cys354 and His336, correspond to Cys442 and His421 in the *E. faecium* homologue. In Ldt_Mt2_ the active site is located under a lid formed by a pair of antiparallel β-strands and their connecting loop (residues 298–324) that protrude from the core of the catalytic domain (Fig. 3 ▶). This feature is not present in the *B.subtilis* enzyme and the tip of the lid is disordered in the *E. faecium* protein, making the active site solvent-accessible, while in Ldt_Mt2_ access is limited by the lid. The large hydrophobic residues of the lid (Tyr298, Tyr308 and Tyr318) and the residues surrounding the entrance to the active site (Tyr330, Phe334 and Trp340) contribute to the closure and to the exclusion of solvent, which may support efficient catalysis.

Residues 382–408 form a C-terminal extension which is not present in the two characterized structural homologues. This sequence stretch runs along the C domain and contributes to the domain interface by forming additional contacts with the B domain (Fig. 1 ▶ *b*). The three tryptophan residues (Trp394, Trp398 and Trp401) in this segment form extensive stacking interactions at the domain interface and are likely to stabilize the relative orientation of the two domains. The pattern of tryptophan residues in this sequence stretch is retained in Rv0483 (LprQ), one of the homologues from the *M. tuberculosis* H37Rv genome.

As has been noted previously (Erdemli *et al.*, 2012 ▶), residues 150–250 (here denoted domain B) fold into an antiparallel β-sandwich (Fig. 1 ▶ *b*) related to the immunoglobulin-like fold (see also below).

### 3.3. The structure of the AB module of Ldt_Mt2_   

The structure of the segment comprising the A and B domains was solved to 1.45 Å resolution by molecular replacement using the B domain (residues 149–250) as the search model. The structure of the AB module includes residues 57–250, approximately half of the extramembrane part of Ldt_Mt2_, and 309 water molecules. Apart from the first two residues of the construct, the electron density for the polypeptide chain is well defined (Fig. 2 ▶ *b*). Seven residues, Val59, Val66, Asn121, Arg123, Thr138, Glu162 and His214, were modelled in double conformations. Each domain in the model contains a bound sulfate ion located on the protein surface. In domain A the sulfate is anchored to the protein through interactions with Asn95, Asn97, Arg99 and Arg122, whereas in the B domain the ligand forms hydrogen bonds to the side chains of Tyr201, Arg209 and Arg211.

**Figure 4:**
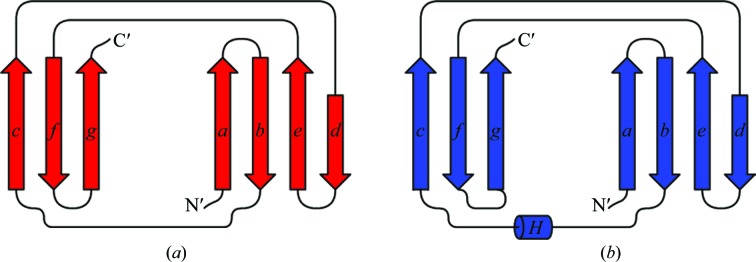
Topology diagrams of the immunoglobulin-like domains in LdtMt2. (*a*) Topology of the A domain. (*b*) Topology of the B domain.

The two domains are arranged in a V-­shaped manner and both display a variant of the immunoglobulin fold built up by a β-sandwich of two antiparallel β-sheets (Figs. 1 ▶ *c* and 4 ▶). A structural classification of immunoglobulin-like β-­sandwich domains defined four subclasses, s-type, h-type, c-type and v-­type, based on strand connectivity and topology (Bork *et al.*, 1994 ▶). Both immunoglobulin-like domains of Ldt_Mt2_ belong to the c-type Ig fold, with strand orders *a*–*b*–*e*–*d* and *c*–­*f*–*g* in the two antiparallel β-sheets (Fig. 4 ▶).

The similarity between the A and B domains is reflected in the C^α^ r.m.s.d. of 2.7 Å based on 85 aligned residues. The main difference in their overall structure is the short α-helix (residues 180–187) found between β-strands β2 and β3, which is not present in domain A, and the loop connecting β6 and β7, which is substantially longer in the B domain (Figs. 4 ▶ and 5 ▶ *a*). The sequence identity derived from structural alignment of the two domains is 12% which does not provide strong evidence for gene duplication.

The domain interface accounts for 350 Å^2^ of buried surface area including 13 and ten buried residues from the A and B domains, respectively. The domain interface is formed by the loops connecting β-strands β1 and β2 and β-­strands β5 and β6 in domain A and the loop connecting β-strands β6 and β7 in domain B. The two domains are connected by a short linker Ala149-His150 forming hydrogen bonds to the main chain of the B-­domain residues involved in the interface (Gly236 and Phe237). The size of the AB-domain interface, the short linker and the fact that *B* factors of the linker residues are in the range 18–22 Å^2^ as in the remaining part of the structure suggest that the domain linker is not flexible and the orientation of the two domains is kept in this V-shaped form (Fig. 1 ▶ *c*). The structures of the B domain in the AB and BC modules are essentially identical and superposition gives an r.m.s.d. of 0.4 Å for 101 aligned residues, although the *B*-factor profiles for these domains are different in the two crystal forms, most likely owing to different crystal-packing inter­actions.

The two constructs used in the crystal structure analysis cover approximately the whole length of the extramembrane part of the polypeptide chain of Ldt_Mt2_ and the results presented here redefine the domain composition of this enzyme. A recent structure analysis described the extramembrane part of the protein as a two-domain structure extending from the membrane (Erdemli *et al.*, 2012 ▶). The structure of the AB module, which covers a larger proportion of the N-terminal residues of the extramembrane part of the enzyme (residues 55–250) than in the previous analysis (residues 120–408), shows that this segment of Ldt_Mt2_ contains an additional domain, domain A, at the N-­terminus (Fig. 1 ▶ *c*) which had not been recognized previously. The extramembrane part of the polypeptide chain of Ldt_Mt2_ is thus a three-domain entity, with two immuno­globulin-like domains rather than one and a C-­terminal transpeptidase domain.

**Figure 5:**
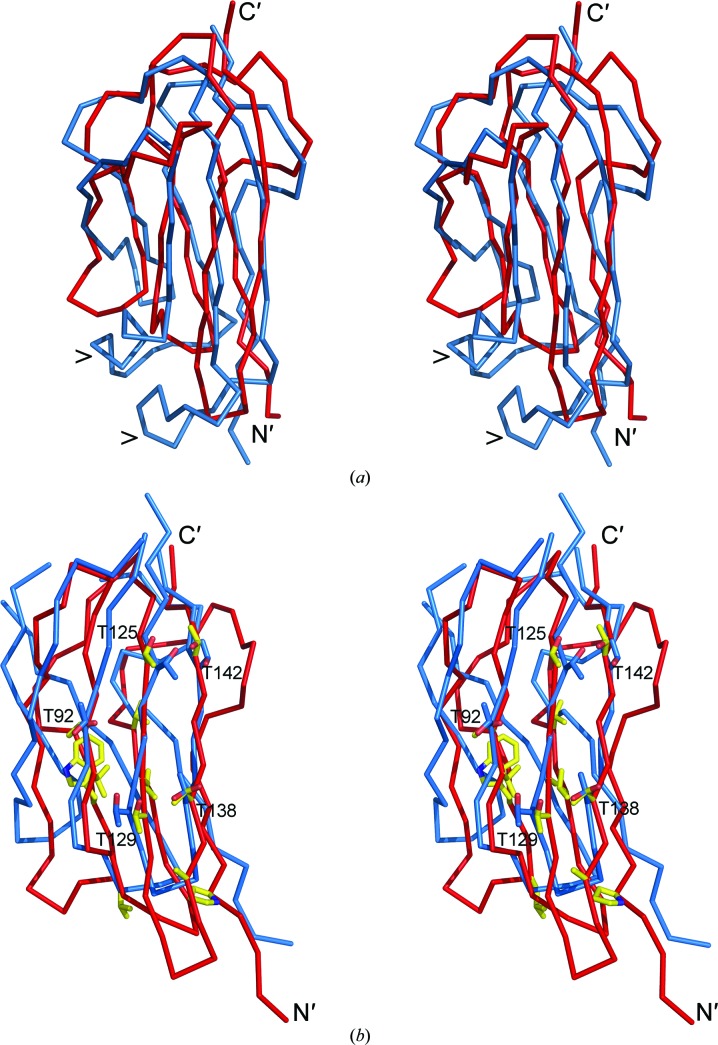
(*a*) Superposition of the A domain (red) and B domain (blue) of Ldt_Mt2_. The major differences in the core fold (indicated by >) are the α-helix (residues 180–187) inserted between β-strands β2 and β3, which is not present in the A domain, and the longer loop connecting β6 and β7 in the B domain. (*b*) Superposition of domain A (red) from Ldt_Mt2_ with the homologous domain of the endo-β-1,4-mannanase from *C. fimi* (light blue; PDB entry 2x2y). Conserved residues of Ldt_Mt2_ are shown as yellow stick models. Five threonine side chains on the surface of the three-stranded β-sheet show a similar pattern in the mannanase and the A domain of Ltd_Mt2_ and are shown as blue sticks.

A structural comparison using *DALI* identified the closest structural homologues of the two immunoglobulin-like domains as the periplasmic copper-resistance protein from *Pseudomonas syringae* (PDB entry 2c9r; Zhang *et al.*, 2006 ▶; *Z*-­score of 10.2 and C^α^ r.m.s.d. of 2.4 Å) and the N-terminal domain of *Halothermothrix orenii* amylase (PDB entry 3bc9; Tan *et al.*, 2008 ▶) and a similar domain of the endo-β-1,4-mannanase from *Cellulomonas fimi* (PDB entry 2x2y; Hekmat *et al.*, 2010 ▶) with similar *Z*-­scores (6.0–10.1) and C^α^ r.m.s.d. values (2.6–3.1 Å). Finally, the cell-wall-located S-­layer protein from *Geobacillus stearo­thermophilus* (PDB entry 2ra1; Pavkov *et al.*, 2008 ▶) aligned with a *Z*-score of 7.8 and a C^α^ r.m.s.d. value of 3.2 Å. Structure-based sequence alignments show that in the case of the B domain the sequence identities to these structural homologues are all below 10%, thus indicating no significant relationship at the amino-acid sequence level. However, the A domain shows a higher degree of amino-acid sequence conservation when compared with the copper-resistance protein from *P. syringae* (18.5%) and with the homologous domains of the mannanase (18.5%) and a glucodextranase from *Arthrobacter globiformis* (15.2%) (Mizuno *et al.*, 2004 ▶). Functional analogy can be excluded in the case of the periplasmic copper-resistance protein, as the copper-binding residues are not present in Ldt_Mt2_. However, the similarity to the two domains that are present in the enzymes processing high-molecular-weight polysaccharides (mannanase and glucodextranase) could potentially be relevant as the PG backbone is a duplex of carbohydrate chains. In the multi-domain amylase enzyme from *H. orenii* it has been experimentally demonstrated that the small noncatalytic immunoglobulin-like domain mediates the interaction of the enzyme with the high-molecular-weight substrate (Tan *et al.*, 2008 ▶). The residues identified as conserved between domain A and the corresponding domains from the polysaccharide-processing enzymes mannanase or glucodextranase are either part of the hydrophobic core or are located on the β-sheet surface (Fig. 5 ▶ *b*).

### 3.4. The extramembrane Ldt_Mt2_   

**Figure 6:**
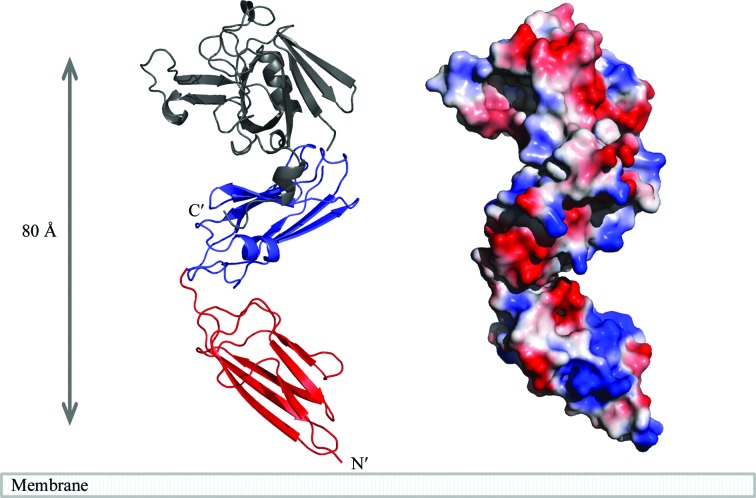
Model of the complete extramembrane part of Ldt_Mt2_ from *M. tuberculosis*. The model was derived by superposition of the B domains common to the structures of the AB and BC modules. The maximal distance between the membrane and the catalytic site, where cross-link formation in the cell-wall peptidoglycan by Ldt_Mt2_ can occur, can be approximated to at least 80–100 Å.

As the two constructs include essentially the whole sequence of the extramembrane part of Ldt_Mt2_ and contain an overlapping domain (domain B), the structure of the full-length protein excluding the short intracellular and membrane-stretch parts can be reconstructed by superposition of the AB and the BC modules based on the B domains (Fig. 6 ▶). The three-domain structure of this transpeptidase can extend from the plasma membrane to a distance of at least 80 Å (plus the stretch of residues 34–54). The remaining four transpeptidase domain proteins in the *M. tuberculosis* H37Rv genome show differences in the length of the polypeptide chain. Rv0116c and Rv1433 both have a predicted transmembrane domain, but their extramembrane segment is shorter at approximately 250 residues and shows significant sequence identity (45 and 38%, respectively) to the BC module of Ldt_Mt2_. On the other hand, Rv0483 and Rv0192 appear to have a three-domain arrangement as in Ldt_Mt2_ as they not only show sequence homology to the B and C domains but also to the A domain (35% sequence identity for Rv0483 and 18% for Rv0192). The five l,d-transpeptidases in the *M. tuberculosis* genome thus can be divided into two groups with one or two immuno­globulin-like domains between the plasma membrane and the catalytic domain. The variation in the number of these domains positions the catalytic domain at different distances from the membrane, reflecting the multilayered structure of the peptidoglycan in the bacterial cell wall. These domains define the distance of the catalytic site and thus the site of formation of the 3–3 linkages in the PG relative to the membrane. Cross-link formation appears to occur at two levels carried out by the BC ­module-like l,d-­transpeptidases (Rv0116c and Rv1433) and by the one domain longer Ldt_Mt2_-type (ABC module; Fig. 6 ▶) enzymes (Rv2518c, Rv0483 and Rv0192). 

### 3.5. Binding of β-lactam antibiotics to Ldt_Mt2_   

The inhibitory effect of β-lactam-type antibiotics towards transpeptidases is based on the formation of a covalent adduct with the active-site serine residues in d,d-transpeptidases or cysteine residues in l,d-transpeptidases, as has been demonstrated for the l,d-transpeptidase from *B. subtilis* (Lecoq *et al.*, 2012 ▶) and for Ldt_Mt1_ (Rv0116c), a homologue of Ldt_Mt2_ (Dubée *et al.*, 2012 ▶). Incubation of the BC module or of the full-length extramembrane fragment (residues 34–408) of Ldt_Mt2_ from *M. tuberculosis* with the β-lactam-type antibiotics imipenem (299.3 Da, carbapenem class) and ampicillin (349.4 Da, penicillin class) led to the formation of covalent enzyme–antibiotic adducts as shown by ESI-MS (Table 2 ▶ and Supplementary Material^1^). The observed molecular masses of the extramembrane fragment (residues 34–408; 40 074.0 Da) and the BC module (residues 149–408; 28 482.0 Da) were very close to the calculated masses from the sequence: 40 073.8 and 28 481.8 Da, respectively (Supplementary Material). The mass difference between the samples before and after incubation with 2 m*M* ampicillin or 2 m*M* imipenem corresponds to the exact mass of the respective antibiotic (Table 2 ▶). Together with the previous demonstration of binding of imipenem and meropenem to Ldt_Mt2_ using ITC (Erdemli *et al.*, 2012 ▶), these data indicate that the enzyme can recognize and bind a variety of β-lactam antibiotics, which also suggests that there is potential for the design of more specific inhibitors of Ldt_Mt2_.

## Supplementary Material

PDB reference: Ltd_Mt2_, AB module, 4hu2

PDB reference: BC module, 4huc

Supporting information file. DOI: 10.1107/S0907444912049268/wd5198sup1.pdf

## Figures and Tables

**Table 1:** Data-collection and refinement statistics Values in parentheses are for the highest resolution shell.

	AB module (57250)	BC module (149408)	BC module (Se-SAD)
PDB code	4hu2	4huc	
Beamline	ID14-1, ESRF	ID14-1, ESRF	ID29, ESRF
Detector	ADSC Q210	ADSC Q210	Pilatus 6M
No. of images	180	180	1800
Oscillation interval ()	1.0	1.0	0.1
Mosaicity ()	0.57	0.63	0.15
Space group	*C*2	*I*2_1_2_1_2_1_	*I*2_1_2_1_2_1_
Unit-cell parameters			
*a* ()	114.4	116.0	116.1
*b* ()	27.6	121.4	121.8
*c* ()	68.0	123.3	124.5
= ()	90.0	90.0	90.0
()	114.1	90.0	90.0
Wavelength ()	0.93340	0.93340	0.97677
Resolution ()	33.71.45 (1.531.45)	58.01.86 (1.961.86)	50.02.75 (2.902.75)
Total No. of reflections	158723 (19668)	514550 (50207)	153521 (22186)
No. of unique reflections	34254 (4636)	71549 (9227)	23295 (3369)
*I*/(*I*)	21.6 (2.8)	23.7 (3.1)	13.7 (2.6)
Multiplicity	4.6 (4.2)	7.2 (5.4)	6.6 (6.6)
Completeness (%)	98.9 (94.1)	98.1 (87.6)	99.9 (99.9)
*R*_merge_ (%)	3.8 (48.9)	6.5 (52.4)	10.5 (82.2)
Wilson *B* value (^2^)	17.6	22.3	74.9
CC_anom			0.423 (0.29)
Refinement			
*R*_cryst_ (%)	16.2	16.5	
*R*_free_ (%)	21.8	18.7	
No. of atoms/*B* factor (^2^)			
Overall	1818/20.3	4570/24.9	
Protein	1499/17.8	4040/23.7	
Ligands	10/31.2 [sulfate]	50/44.7 [acetate, Na^+^]	
Water	309/32.1	480/32.8	
R.m.s.d. from ideal geometry			
Bond lengths ()	0.009	0.013	
Bond angles ()	1.44	1.48	
Ramachandran plot (%)			
Residues in preferred regions	98.3	95.9	
Residues in allowed regions	1.7	4.1	
Outliers	0	0	

**Table 2:** Covalent adducts formed by Ldt_Mt2_ from *M. tuberculosis* with the -lactam-type antibiotics imipenem and ampicillin

Protein construct, molecular mass (Da)	-Lactam, molecular mass (Da)	Detected mass (Da)	Mass difference (Da)
Ldt_Mt2_ periplasmic segment (34408), 40074.0	Imipenem, 299.3	40374.6	300.6
Ldt_Mt2_ BC module (149408), 28482.0	Imipenem, 299.3	28780.9	298.9
	Ampicillin, 349.4	28831.7	349.7

## Footnotes

1 Supplementary material has been deposited in the IUCr electronic archive (Reference: WD5198). Services for accessing this material are described at the back of the journal.

## References

[bb1] Altschul, S. F., Gish, W., Miller, W., Myers, E. W. & Lipman, D. J. (1990). *J. Mol. Biol.* **215**, 403–410. 10.1016/S0022-2836(05)80360-2 2231712

[bb2] Biarrotte-Sorin, S., Hugonnet, J.-E., Delfosse, V., Mainardi, J.-L., Gutmann, L., Arthur, M. & Mayer, C. (2006). *J. Mol. Biol.* **359**, 533–538. 10.1016/j.jmb.2006.03.014 16647082

[bb3] Bielnicki, J., Devedjiev, Y., Derewenda, U., Dauter, Z., Joachimiak, A. & Derewenda, Z. S. (2006). *Proteins*, **62**, 144–151. 10.1002/prot.20702 PMC2792008 16287140

[bb4] Bork, P., Holm, L. & Sander, C. (1994). *J. Mol. Biol.* **242**, 309–320. 10.1006/jmbi.1994.1582 7932691

[bb5] Brennan, P. J. & Crick, D. C. (2007). *Curr. Top. Med. Chem.* **7**, 475–488. 10.2174/156802607780059763 17346193

[bb6] Cava, F., de Pedro, M. A., Lam, H., Davis, B. M. & Waldor, M. K. (2011). *EMBO J.* **30**, 3442–3453. 10.1038/emboj.2011.246 PMC3160665 21792174

[bb7] Chen, V. B., Arendall, W. B., Headd, J. J., Keedy, D. A., Immormino, R. M., Kapral, G. J., Murray, L. W., Richardson, J. S. & Richardson, D. C. (2010). *Acta Cryst.* D**66**, 12–21. 10.1107/S0907444909042073 PMC2803126 20057044

[bb8] Cole, C., Barber, J. D. & Barton, G. J. (2008). *Nucleic Acids Res.* **35**, W197–W201. 10.1093/nar/gkn238 PMC2447793 18463136

[bb9] Cole, S. T. & Riccardi, G. (2011). *Curr. Opin. Microbiol.* **14**, 570–576. 10.1016/j.mib.2011.07.022 21821466

[bb10] Cowtan, K. (2010). *Acta Cryst.* D**66**, 470–478. 10.1107/S090744490903947X PMC2852311 20383000

[bb11] Cserzö, M., Wallin, E., Simon, I., von Heijne, G. & Elofsson, A. (1997). *Protein Eng.* **10**, 673–676. 10.1093/protein/10.6.673 9278280

[bb12] Doublié, S. (1997). *Methods Enzymol.* **276**, 523–530. 9048379

[bb13] Dubée, V., Triboulet, S., Mainardi, J.-L., Ethève-Quelquejeu, M., Gutmann, L., Marie, A., Dubost, L., Hugonnet, J.-E. & Arthur, M. (2012). *Antimicrob. Agents Chemother.* **56**, 4189–4195. 10.1128/AAC.00665-12 PMC3421625 22615283

[bb14] Ebina, T., Toh, H. & Kuroda, Y. (2009). *Biopolymers*, **92**, 1–8. 10.1002/bip.21105 18844295

[bb15] Emsley, P., Lohkamp, B., Scott, W. G. & Cowtan, K. (2010). *Acta Cryst.* D**66**, 486–501. 10.1107/S0907444910007493 PMC2852313 20383002

[bb16] England, K., Boshoff, H. I., Arora, K., Weiner, D., Dayao, E., Schimel, D., Via, L. E. & Barry, C. E. III (2012). *Antimicrob. Agents Chemother.* **56**, 3384–3387. 10.1128/AAC.05690-11 PMC3370771 22450968

[bb17] Erdemli, S. B., Gupta, R., Bishai, W. R., Lamichhane, G., Amzel, L. M. & Bianchet, M. A. (2012). *Structure*, **20**, 2103–2115. 10.1016/j.str.2012.09.016 PMC3573878 23103390

[bb18] Flores, A. R., Parsons, L. M. & Pavelka, M. S. (2005). *Microbiology*, **151**, 521–532. 10.1099/mic.0.27629-0 15699201

[bb19] Gengenbacher, M. & Kaufmann, S. H. (2012). *FEMS Microbiol. Rev.* **36**, 514–532. 10.1111/j.1574-6976.2012.00331.x PMC3319523 22320122

[bb20] Gupta, R., Lavollay, M., Mainardi, J.-L., Arthur, M., Bishai, W. R. & Lamichhane, G. (2010). *Nature Med.* **16**, 466–469. 10.1038/nm.2120 PMC2851841 20305661

[bb21] Hekmat, O., Lo Leggio, L., Rosengren, A., Kamarauskaite, J., Kolenova, K. & Stålbrand, H. (2010). *Biochemistry*, **49**, 4884–4896. 10.1021/bi100097f 20426480

[bb22] Hett, E. C. & Rubin, E. J. (2008). *Microbiol. Mol. Biol. Rev.* **72**, 126–156. 10.1128/MMBR.00028-07 PMC2268284 18322037

[bb23] Holm, L. & Park, J. (2000). *Bioinformatics*, **16**, 566–567. 10.1093/bioinformatics/16.6.566 10980157

[bb24] Holm, L. & Rosenström, P. (2010). *Nucleic Acids Res.* **38**, W545–W549. 10.1093/nar/gkq366 PMC2896194 20457744

[bb25] Hugonnet, J.-E. & Blanchard, J. S. (2007). *Biochemistry*, **46**, 11998–12004. 10.1021/bi701506h PMC2593862 17915954

[bb26] Hugonnet, J.-E., Tremblay, L. W., Boshoff, H. I., Barry, C. E. III & Blanchard, J. S. (2009). *Science*, **323**, 1215–1218. 10.1126/science.1167498 PMC2679150 19251630

[bb27] Kabsch, W. (2010). *Acta Cryst.* D**66**, 125–132. 10.1107/S0907444909047337 PMC2815665 20124692

[bb28] Krissinel, E. & Henrick, K. (2007). *J. Mol. Biol.* **372**, 774–797. 10.1016/j.jmb.2007.05.022 17681537

[bb29] Kumar, P., Arora, K., Lloyd, J. R., Lee, I. Y., Nair, V., Fischer, E., Boshoff, H. I. & Barry, C. E. III (2012). *Mol. Microbiol.* **86**, 367–381. 10.1111/j.1365-2958.2012.08199.x PMC3468717 22906310

[bb30] Lavollay, M., Arthur, M., Fourgeaud, M., Dubost, L., Marie, A., Veziris, N., Blanot, D., Gutmann, L. & Mainardi, J.-L. (2008). *J. Bacteriol.* **190**, 4360–4366. 10.1128/JB.00239-08 PMC2446752 18408028

[bb31] Lecoq, L., Bougault, C., Hugonnet, J.-E., Veckerlé, C., Pessey, O., Arthur, M. & Simorre, J.-P. (2012). *Structure*, **20**, 850–861. 10.1016/j.str.2012.03.015 22579252

[bb51] Leslie, A. G. W. (2006). *Acta Cryst.* D**62**, 48–57. 10.1107/S0907444905039107 16369093

[bb33] Lovering, A. L., Safadi, S. S. & Strynadka, N. C. (2012). *Annu. Rev. Biochem.* **81**, 451–478. 10.1146/annurev-biochem-061809-112742 22663080

[bb34] Mainardi, J.-L., Fourgeaud, M., Hugonnet, J.-E., Dubost, L., Brouard, J.-P., Ouazzani, J., Rice, L. B., Gutmann, L. & Arthur, M. (2005). *J. Biol. Chem.* **18**, 38146–38152. 10.1074/jbc.M507384200 16144833

[bb35] McCoy, A. J., Grosse-Kunstleve, R. W., Adams, P. D., Winn, M. D., Storoni, L. C. & Read, R. J. (2007). *J. Appl. Cryst.* **40**, 658–674. 10.1107/S0021889807021206 PMC2483472 19461840

[bb36] Meroueh, S. O., Bencze, K. Z., Hesek, D., Lee, M., Fisher, J. F., Stemmler, T. L. & Mobashery, S. (2006). *Proc. Natl Acad. Sci. USA*, **103**, 4404–4409. 10.1073/pnas.0510182103 PMC1450184 16537437

[bb37] Mizuno, M., Tonozuka, T., Suzuki, S., Uotsu-Tomita, R., Kamitori, S., Nishikawa, A. & Sakano, Y. (2004). *J. Biol. Chem.* **279**, 10575–10583. 10.1074/jbc.M310771200 14660574

[bb38] Mooij, W. T., Cohen, S. X., Joosten, K., Murshudov, G. N. & Perrakis, A. (2009). *Structure*, **17**, 183–189. 10.1016/j.str.2008.12.011 PMC2670983 19217389

[bb39] Murshudov, G. N., Skubák, P., Lebedev, A. A., Pannu, N. S., Steiner, R. A., Nicholls, R. A., Winn, M. D., Long, F. & Vagin, A. A. (2011). *Acta Cryst.* D**67**, 355–367. 10.1107/S0907444911001314 PMC3069751 21460454

[bb40] Pavkov, T., Egelseer, E. M., Tesarz, M., Svergun, D. I., Sleytr, U. B. & Keller, W. (2008). *Structure*, **16**, 1226–1237. 10.1016/j.str.2008.05.012 18682224

[bb41] Rahman, O., Cummings, S. P., Harrington, D. J. & Sutcliffe, I. C. (2008). *World J. Microbiol. Biotechnol.* **24**, 2377–2382.

[bb42] Russell, D. G. (2011). *Immunol. Rev.* **240**, 252–268. 10.1111/j.1600-065X.2010.00984.x PMC3174472 21349098

[bb44] Sheldrick, G. M. (2008). *Acta Cryst.* A**64**, 112–122. 10.1107/S0108767307043930 18156677

[bb45] Sundqvist, G., Stenvall, M., Berglund, H., Ottosson, J. & Brumer, H. (2007). *J. Chromatogr. B Analyt. Technol. Biomed. Life Sci.* **852**, 188–194. 10.1016/j.jchromb.2007.01.011 17267305

[bb46] Tan, T.-C., Mijts, B. N., Swaminathan, K., Patel, B. K. & Divne, C. (2008). *J. Mol. Biol.* **378**, 852–870. 10.1016/j.jmb.2008.02.041 18387632

[bb47] Thompson, J. D., Higgins, D. G. & Gibson, T. J. (1994). *Nucleic Acids Res.* **22**, 4673–4680. 10.1093/nar/22.22.4673 PMC308517 7984417

[bb48] Vollmer, W., Blanot, D. & de Pedro, M. A. (2008). *FEMS Microbiol. Rev.* **32**, 149–167. 10.1111/j.1574-6976.2007.00094.x 18194336

[bb49] Winn, M. D. *et al.* (2011). *Acta Cryst.* D**67**, 235–242.

[bb50] Zhang, L., Koay, M., Maher, M. J., Xiao, Z. & Wedd, A. G. (2006). *J. Am. Chem. Soc.* **128**, 5834–5850. 10.1021/ja058528x 16637653

