# Supplementary material for: Structure of LdtMt2, an l,d-transpeptidase from Mycobacterium tuberculosis
Source: Acta Crystallogr D Biol Crystallogr. 2013 Feb 16;69(Pt 3):432–41. doi: 10.1107/S0907444912049268 (PMC3605044; doi:10.1107/S0907444912049268)

Figure S1: (A) Mass spectrometric analysis of the Ldt_{Mt2} segment (residues 34-408) with and without imipinem (300.6 Da). The mass difference in the major peaks corresponds very closely to the mass of imipinem (299.3 Da). (B) Molecular mass of the BC-module (residues 149-408), 28481.7 Da. The increase in mass upon binding β-lactam antibiotics, imipinem (299.3 Da) and ampicillin (349.4 Da) could be validated with 28780.1 Da and 28831.7 Da for each covalently formed adduct respectively.

A

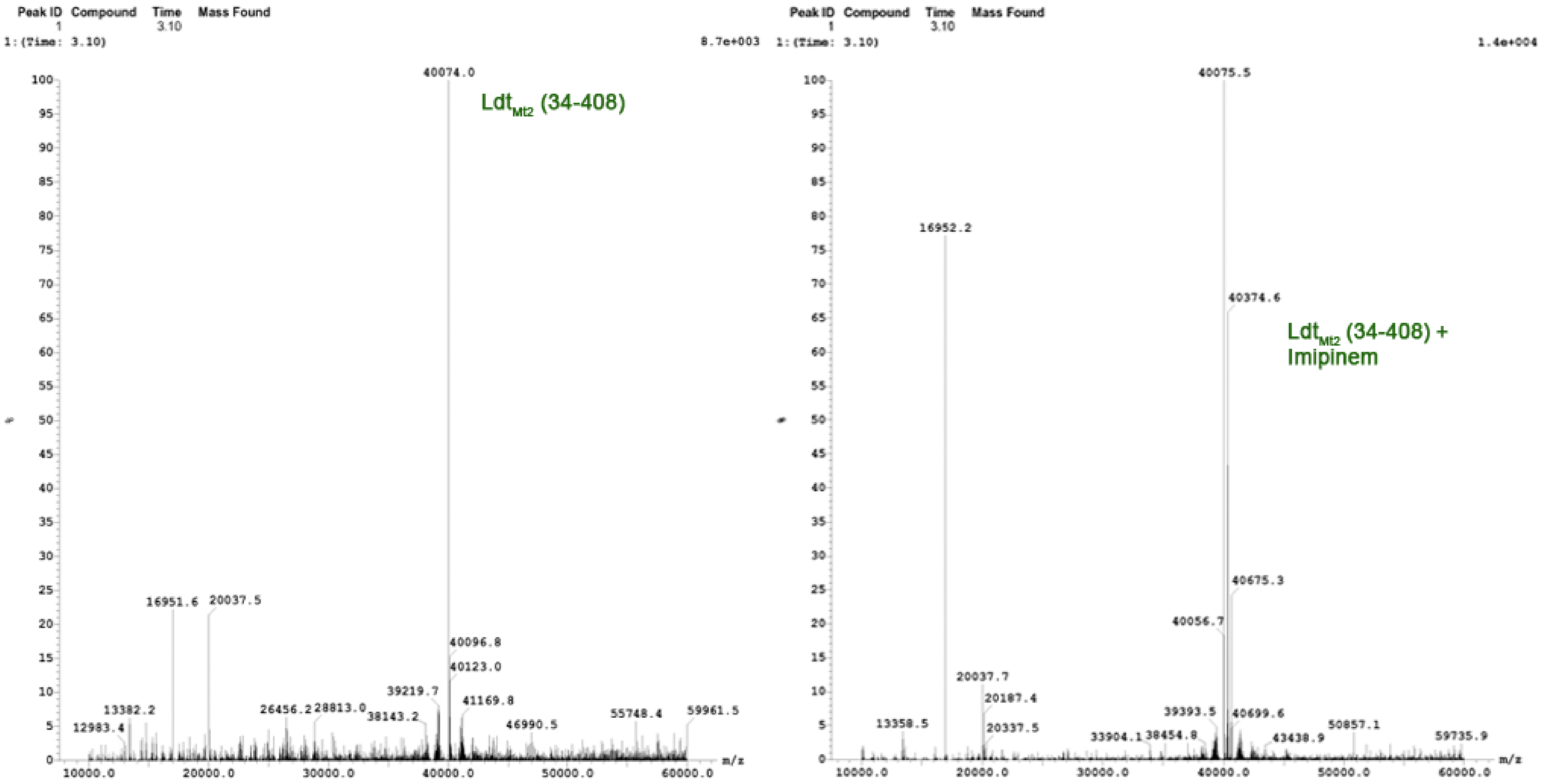

Peak ID Compound Time Mass Found
1 3.10
1: (Time: 3.10)
8.7e+003
40074.0
Ldt$_{Mt2}$ (34-408)
16951.6
20037.5
40096.8
40123.0
39219.7
41169.8
12983.4
13382.2
26456.2
28813.0
38143.2
46990.5
55748.4
59961.5
%
m/z
10000.0
20000.0
30000.0
40000.0
50000.0
60000.0
Peak ID Compound Time Mass Found
1 3.10
1: (Time: 3.10)
1.4e+004
40075.5
16952.2
40374.6
Ldt$_{Mt2}$ (34-408) +
Imipenem
40675.3
40056.7
20037.7
20187.4
20337.5
13358.5
33904.1
38454.8
39393.5
40699.6
43438.9
50857.1
59735.9

B

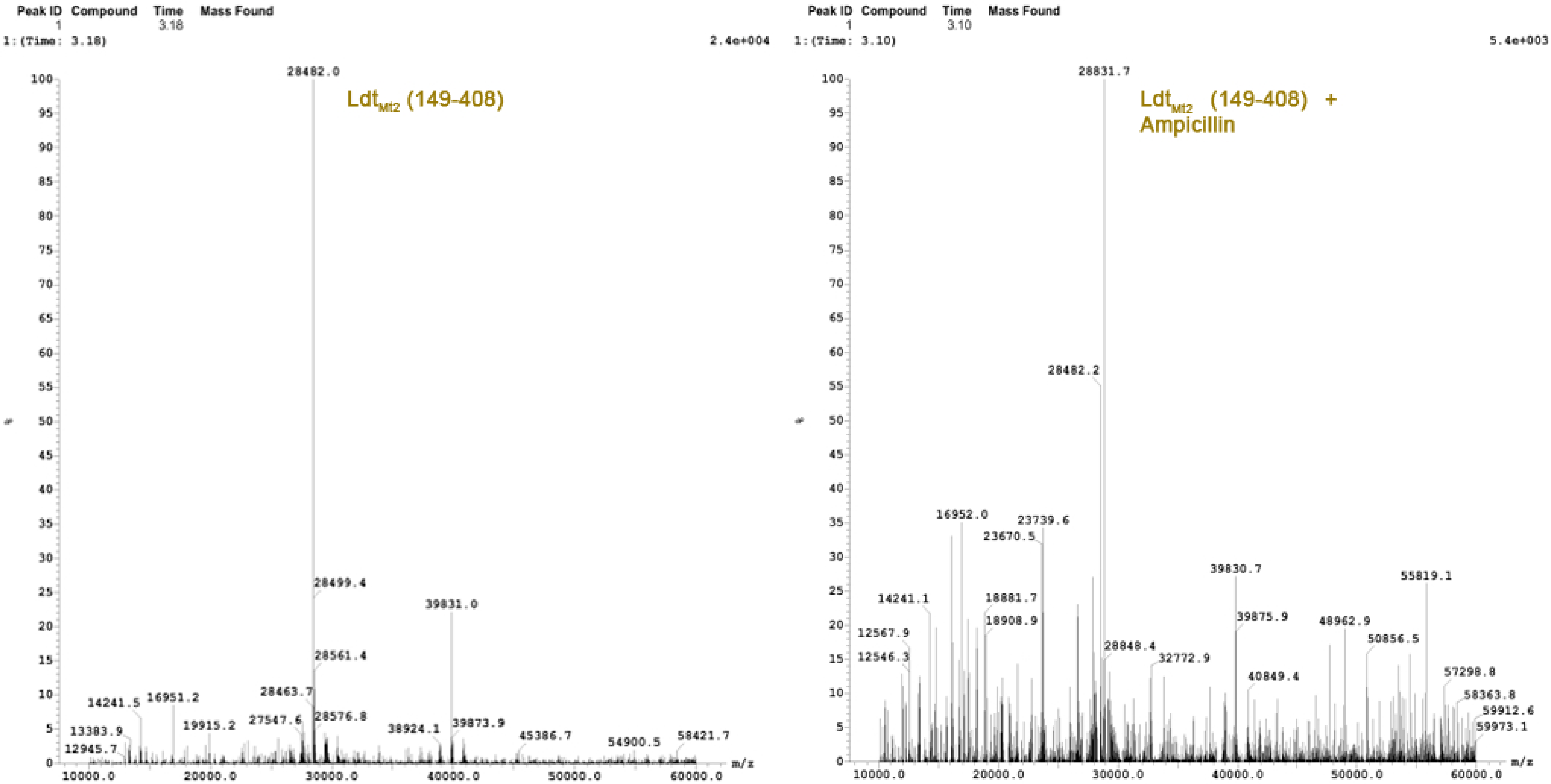

Peak ID Compound Time Mass Found
1 3.18
1: (Time: 3.18)
2.4e+004
Ldt$_{Mt2}$ (149-408)
28482.0
28499.4
28561.4
28463.7
28576.8
27547.6
39831.0
39873.9
38924.1
14241.5
16951.2
19915.2
13383.9
12945.7
45386.7
54900.5
58421.7
%
m/z
Peak ID Compound Time Mass Found
1 3.10
1: (Time: 3.10)
5.4e+003
Ldt$_{Mt2}$ (149-408) + Ampicillin
28831.7
28482.2
16952.0
23739.6
23670.5
14241.1
18881.7
18908.9
12567.9
12546.3
28848.4
32772.9
39830.7
39875.9
40849.4
48962.9
50856.5
55819.1
57298.8
58363.8
59912.6
59973.1
%
m/z

Peak ID	Compound	Time	Mass Found
1		3.10	

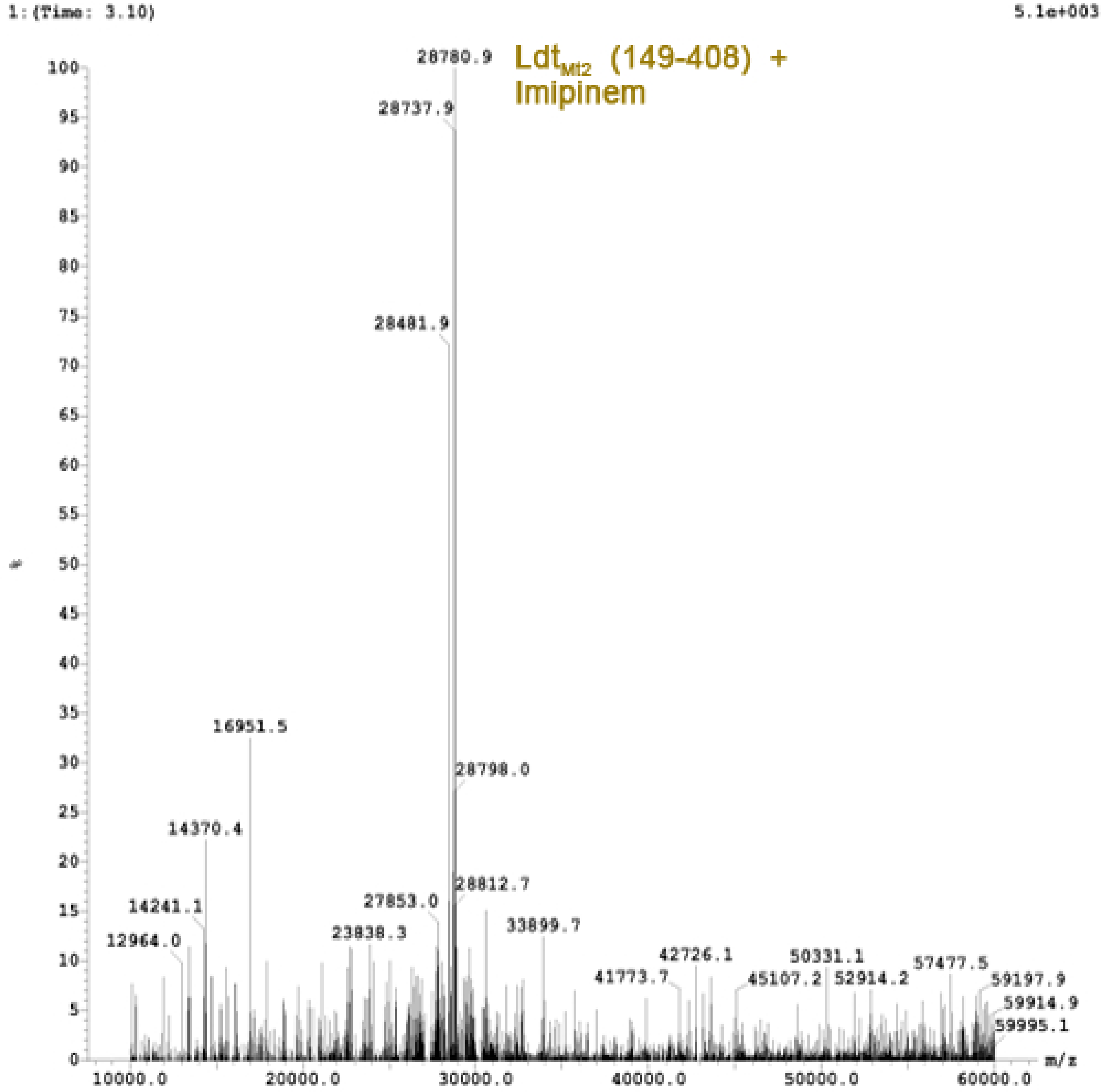

Supplement: Supplementary file 1 [file d-69-00432-sup1.pdf]
